# Prevalence of burnout and its risk and protective factors among healthcare workers in the Middle East, North Africa, and Turkey: a systematic review and meta-analysis

**DOI:** 10.3389/fpsyg.2025.1539105

**Published:** 2025-10-22

**Authors:** Mohammed A. Alhassan, Mohammed A. Alarabi, Shaden Abdulrahman Alhassan, Waled M. Albalawi, Ebraheem S. AlRabiah

**Affiliations:** ^1^Department of Medical Specialties, College of Medicine, Majmaah University, Majmaah, Saudi Arabia; ^2^Department of Psychiatry, King Saud University, Riyadh, Saudi Arabia; ^3^Dar Al Uloom University, Riyadh, Saudi Arabia; ^4^King Salman Armed Forces Hospital, Tabuk, Saudi Arabia; ^5^Special Security Forces Healthcare Center, Riyadh, Saudi Arabia

**Keywords:** burnout, healthcare workers, work stress, MBI-HSS, MENAT region, emotional exhaustion, depersonalization, personal accomplishment

## Abstract

**Background:**

Burnout, defined as persistent work-related stress, is a critical concern among healthcare workers (HCWs), particularly in high-demand environments such as the Middle East, North Africa, and Turkey (MENAT) region. This systematic review and meta-analysis aimed to assess the pooled prevalence of burnout and examine its associated risk and protective factors among HCWs in the MENAT context.

**Methods:**

A comprehensive search of studies published between 2013 and 2024 identified 123 studies involving 36,769 participants. Only studies using the Maslach Burnout Inventory - Human Services Survey (MBI-HSS) were included. Burnout was evaluated across its three dimensions: emotional exhaustion (EE), depersonalization (DP), and personal accomplishment (PA). The protocol is registered with PROSPERO (CRD420251051167).

**Results:**

The pooled prevalence was 40% for high emotional exhaustion, 31% for high depersonalization, and 38% for low personal accomplishment. The overall mean scores were 22.02 for EE, 10.07 for DP, and 25.49 for PA. Substantial heterogeneity across studies reflected wide variation in healthcare system capacity and workforce conditions. Burnout was more pronounced in countries such as Saudi Arabia, Iran, and Turkey. Common risk factors included high workload, lack of institutional support, younger age, and female gender. Protective factors were linked to increased autonomy, leadership support, and strong peer relationships.

**Conclusion:**

Burnout is prevalent among HCWs across the MENAT region, with significant variability across countries. Targeted interventions to reduce occupational stressors and enhance protective workplace structures are urgently needed to safeguard provider wellbeing and improve healthcare delivery.

**Systematic review registration:**

https://www.crd.york.ac.uk/prospero/display_record.php?ID=CRD420251051167, identifier CRD420251051167

## Introduction

Burnout is a syndrome characterized by chronic occupational stress, first conceptualized in the early 1970s by psychoanalyst Herbert Freudenberger (Freudenberger, 1974; Maslach, 1998). Burnout is commonly defined by three core dimensions: emotional exhaustion (EE), characterized by mental fatigue and work-related stress; cynicism or depersonalization (DP), marked by a detached, negative attitude toward others in the workplace; and diminished professional efficacy or personal accomplishment (PA) (Maslach, 1998). These dimensions reflect the impact of persistent job-related stress on an individual’s emotional and professional well-being. While burnout can occur in various professions, it is particularly prevalent in healthcare, where high job demands and chronic stress significantly impact workers, particularly among nurses and physicians, where occupational stress is a significant public health concern. Its impact extends beyond individuals, influencing patient safety, healthcare quality, and organizational performance (Yang and Hayes, 2020).

Healthcare workers experiencing burnout may exhibit decreased job satisfaction, higher rates of absenteeism, an increased risk of patient safety incidents, including medical errors (Suñer-Soler et al., 2014; Yang and Hayes, 2020). Recent research shows that burnout in healthcare workers is associated with factors such as excessive workload, time constraints, role ambiguity, limited social support, and restricted decision-making autonomy (De Hert, 2020). Burnout is also linked to serious personal consequences, such as mental health disorders, substance abuse, strained relationships, and even suicidal ideation (Abdelhafiz et al., 2020).

Burnout places a significant economic burden on healthcare systems, contributing to higher workforce turnover, malpractice risks, and increased healthcare costs due to reduced quality of care (De Hert, 2020). Organizational models, including Karasek’s job strain model and the effort-reward imbalance model, highlight how high job demands combined with low control and inadequate rewards contribute to burnout risk (Kain and Jex, 2010; Siegrist, 2016). Addressing these factors is essential to improving healthcare workers’ well-being and ensuring high-quality patient care. Although burnout is widely recognized as a critical occupational issue, especially in healthcare, research in this area continues to face significant challenges (De Hert, 2020). Research in this area is often limited by conceptual overlaps with other psychosocial stress factors and variations in burnout experiences based on gender, age, specialty, and geographic training context (De Hert, 2020).

The Middle East, North Africa, and Turkey (MENAT) region, in particular, represents a culturally diverse The Middle East, North Africa, and Turkey (MENAT) region is culturally diverse, with significant variations in economic development, healthcare systems, and workplace environments. However, research on burnout in this region remains limited. Factors such as cultural attitudes toward mental health, workforce challenges, and disparities in healthcare infrastructure highlight the need for region-specific investigations that account for these socio-cultural and systemic differences (Awad et al., 2022).

Addressing burnout in the MENAT region is essential, as healthcare workforce stability directly impacts public health and socio-economic development. Despite growing global research on burnout, substantial gaps remain in understanding the unique stressors and protective factors affecting healthcare professionals in this region. Given the diverse economic conditions and healthcare disparities across MENAT countries, a deeper exploration of burnout in this context is crucial.

While previous reviews have examined burnout among healthcare workers globally, research specific to the MENAT region remains limited. This study is the first systematic review and meta-analysis to quantify burnout prevalence and its determinants across MENAT countries, identifying region-specific risk and protective factors to inform targeted interventions. This systematic review and meta-analysis examine burnout among healthcare workers in the MENAT region, focusing on its prevalence, risk factors, and protective factors.

## Methods

This systematic review and meta-analysis were conducted in accordance with the Preferred Reporting Items for Systematic Reviews and Meta-Analyses (PRISMA) criteria. These standards were employed to oversee all phases of the research (Page et al., 2021). The review protocol was registered with the International Prospective Register of Systematic Reviews (PROSPERO), registration number CRD420251051167.

### Search strategy

We conducted a systematic search of the literature using Scopus, Medline, Embase, and PsycINFO, supplemented by a manual reference search of included studies to ensure comprehensive coverage. The search spanned January 1, 2013, to December 31, 2024, to capture recent shifts in healthcare work environments and burnout research Search strategy combined keywords and controlled vocabulary related to the core concepts of this review: “burnout,” healthcare workers, and the MENAT region. Synonyms and closely related terms for burnout (e.g., “burnout,” “occupational stress”), for healthcare workers (e.g., “healthcare personnel,” “medical staff,” “nurse,” “physician”), and for the MENAT geographical region (including terms like “Middle East,” “North Africa,” “Turkey,” as well as the names of specific countries in the region) were used. These terms were combined using Boolean operators (OR for synonyms and AND to link the different concept groups) and applied to search within titles, abstracts, and keywords. The full detailed search strings for each database are provided in the Supplementary File 1, ensuring transparency and reproducibility of the search.

### Inclusion and exclusion criteria

Our criteria included published articles in English that are peer-reviewed and conducted in the MENAT region within the specified time period. Exclusion criteria included studies without primary data, non-healthcare populations, and non-English articles unless a translation was available. Any cross-sectional and prevalence studies that assess burnout subscale prevalence, including high EE, high DP, and low PA, or reports detailed scores for these subscales using MBI-Human Services Survey (MBI-HSS) were included in the meta-analysis. Search and selection criteria are detailed in Table 1. Cohort studies and randomized controlled trials (RCTs) were not included, as they primarily focus on interventions or longitudinal outcomes rather than burnout prevalence, which was the focus of this review.

**Table 1:** Inclusion criteria for studies on burnout among healthcare workers in the MENAT region.

Framework	Criteria
Population	Healthcare workers (HCWs). Reports published from January 1st, 2013, to December 31st, 2024. Reports published from the MENAT (Middle East, North Africa and Turkey) region. The region includes the following countries: Algeria Bahrain Djibouti Egypt Iran Iraq Israel Jordan Kuwait Lebanon Libya Morocco Oman Palestine Qatar Saudi Arabia Somalia Sudan Syria Tunisia UAE Yemen Turkey
Interventions	No Intervention
Comparisons or control groups	Comparison group was not examined in this investigation because there was no comparison group.
Outcomes	Prevalence of Burnout calculated using Maslach Burnout Inventory
Study designs	Only the following reports were included: Cross-sectional studies. Prevalence studies.

The MBI-HSS was developed for the human services field and included 22 items; emotional exhaustion (MBI-EE nine items), depersonalization (MBI-DP five items), personal accomplishment (MBI-PA eight items). The scores for each of the three factors are totaled separately and can be coded as low, average or high using cut-off scores defined in the MBI Manual (Maslach and Jackson, 1996). Reliability and validity of the MBI-HSS have been established across a wide range of countries and professional settings including in the mental health field (Gil-Monte, 2005; Kitaoka-Higashiguchi et al., 2004; Maslach and Jackson, 1996; Paris and Hoge, 2010; Poghosyan et al., 2009).

### Selection method

Search results were imported and managed using EndNote 20 (Thomson Reuters, New York, USA). Duplicates were removed electronically and then manually.

The reviewers initially examined and selected articles based on their title and abstract individually. Full text of all the references that met the inclusion criteria were requested. Abstracts were used for articles that lacked full texts and full text could not be retrieved, or if full texts were not available in English. The reviewers then gathered data from the selected articles and removed studies that fit the exclusion criteria. All discrepancies and disagreements among investigators regarding inclusion and exclusion criteria were resolved through unanimous decision.

### Data extraction

Data extraction was conducted independently by two reviewers (M.A. and S.A.) using a standardized data extraction form. In cases of disagreement, a third reviewer (M.A A) was consulted to resolve discrepancies and reach consensus. Extracted data included general and study-specific characteristics: author name, year of publication, country, type and number of healthcare workers (e.g., physicians, nurses), gender distribution, mean age of participants (if reported), the definition and cut-offs used for the MBI-HSS, and mean scores and/or prevalence estimates for emotional exhaustion (EE), depersonalization (DP), and personal accomplishment (PA).

### Quality assessment

Two independent reviewers conducted a quality assessment at both the individual study level and the result level. The Joanna Briggs Institute (JBI) critical appraisal checklist for prevalence studies was employed to evaluate the methodological quality of a study and to ascertain the degree to which the study has mitigated potential bias in its design, execution, and analysis (Munn et al., 2015).

### Statistical analysis

Proportional meta-analyses were conducted to estimate the pooled prevalence of burnout and its dimensions: EE, DP, and reduced PA. Meta-analyses of pooled means were utilized to get the overall EE, DP, and PA subscale scores throughout the MENAT region. Random-effects models were employed to account for potential heterogeneity across studies, considering variations in study populations and methodologies. Heterogeneity was assessed using the I^2^ statistic, with values above 50% indicating moderate to high heterogeneity. Subgroup analyses explored variations in burnout prevalence based on geographical region. Publication bias was assessed through burnout subscale prevalence using funnel plots and Egger’s tests. All statistical analyses, including meta-analyses, forest plots, and funnel plots, were performed using R software version 4.3.1., the “meta” package, evaluating heterogeneity, and generating visualizations.

## Results

### Search outcome and quality assessment

The electronic literature search across Scopus, Medline, Embase, and PsycINFO databases identified a total of 2,405 records. After removing 1,204 duplicates, 1,201 unique records remained for screening. Of these, 1,012 were excluded based on titles and abstracts. Full-text reviews were conducted for 189 reports, of which 66 were excluded for not meeting the inclusion criteria. Ultimately, 123 studies involving 36,769 participants were included in this systematic review. All included studies were peer-reviewed and utilized the Maslach Burnout Inventory - Human Services Survey (MBI-HSS) to assess burnout dimensions.

The studies were geographically distributed across 15 MENAT countries: Egypt, Iran, Jordan, Saudi Arabia, Turkey, Tunisia, Oman, Morocco, Iraq, Sudan, Qatar, Lebanon, Bahrain, Palestine, and Israel. No eligible data using the MBI-HSS were identified from Algeria, Djibouti, Kuwait, Libya, Somalia, Syria, the United Arab Emirates, or Yemen.

**Figure 1:**
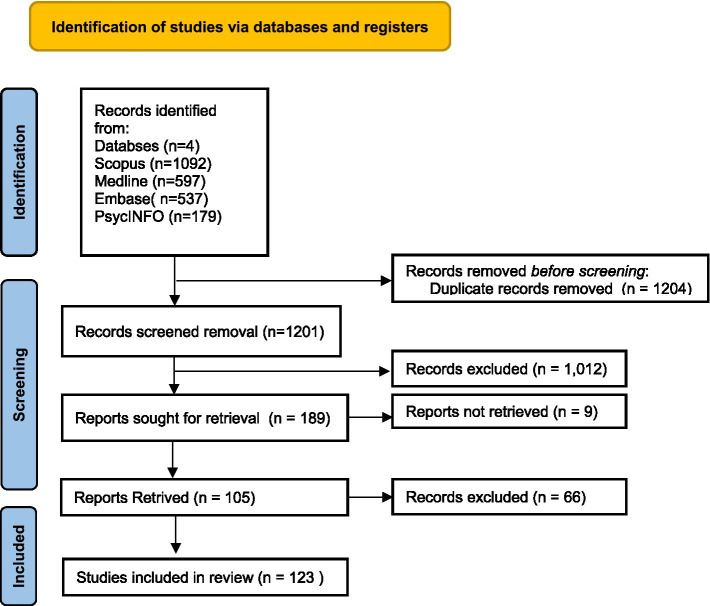
PRISMA flow diagram.

Quality assessment using the JBI critical appraisal checklist indicated that the majority of studies were of moderate to high methodological quality, with consistent application of the MBI-HSS and appropriate statistical analysis. Most studies adequately described their populations and employed standardized measurement procedures. However, common limitations included incomplete reporting of response rates, unclear sampling strategies, and limited justification of sample sizes. These methodological concerns may introduce bias and should be considered when interpreting the pooled findings. Full appraisal results are available in Supplementary File 2 and Figure 1.

### Study population

Studies conducted across MENAT were identified. All studies used self-reported questionnaires. The number of respondents ranged from 30 (Chahbounia et al., 2023) to 2,592 (Bazmi et al., 2019; Topçu et al., 2016). Female respondents were over-represented, as the pooled females responders reached 60.53% (95% CI: 54.36, 66.39). Oman was the country that included the highest female prevalence, 99.09%, in contrast to Bahrain, that included the lowest prevalence of females 39.3%. The mean age of respondents was 34.25 (95% CI: 33.09, 35.41) years. Morocco reported responders with the highest average age groups, 45.73 years, in contrast to Egypt, which contained the lowest average ages, 29.8. Characteristics of all Included Studies on Burnout Among Healthcare Workers in the MENAT Region are summarized in the Supplementary File 3.

### Mean scores on burnout subscales

**Figure 2:**
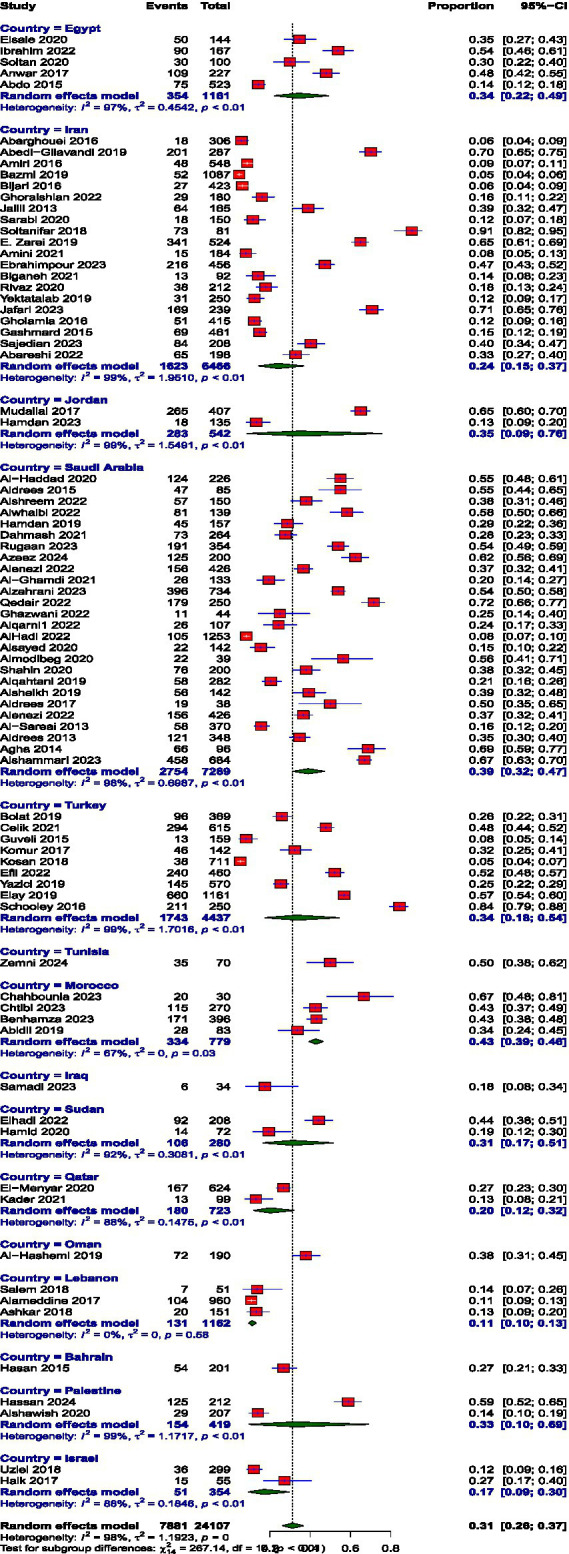
Prevalence of depersonalization.

**Figure 3:**
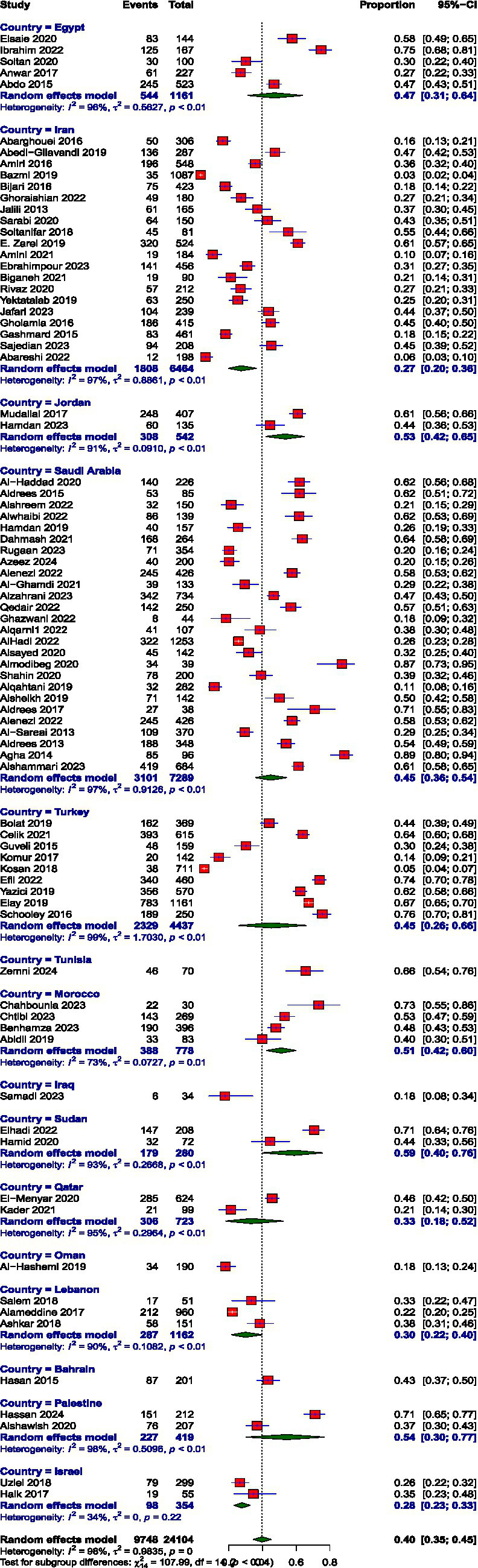
Prevalence of emotional exhaustion.

**Figure 4:**
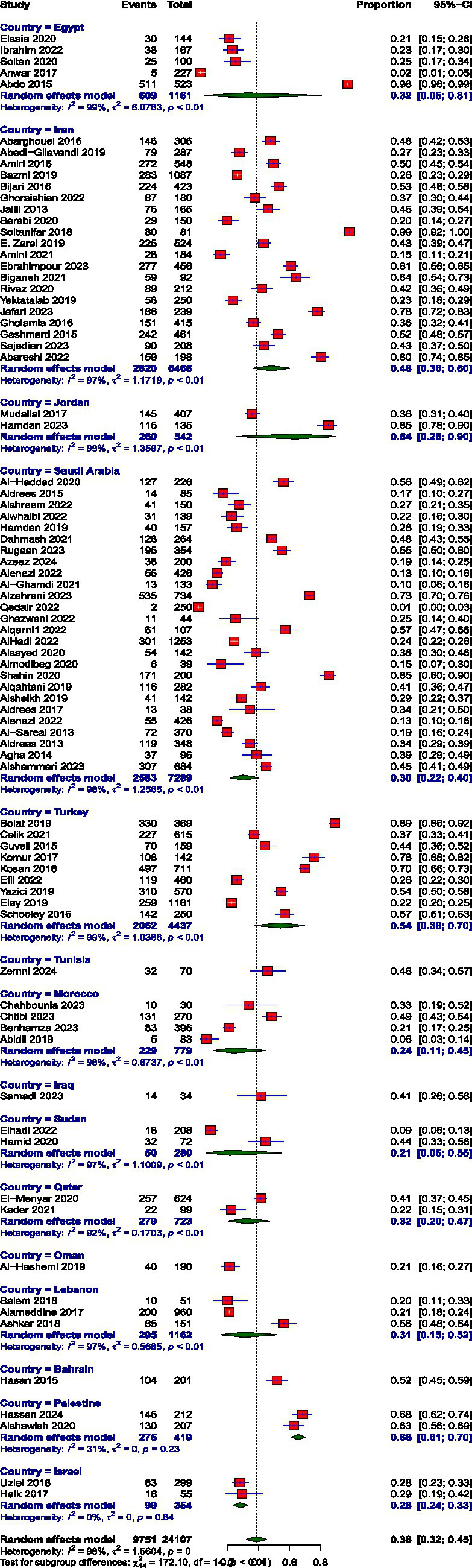
Prevalence of personal accomplishment.

**Figure 5:**
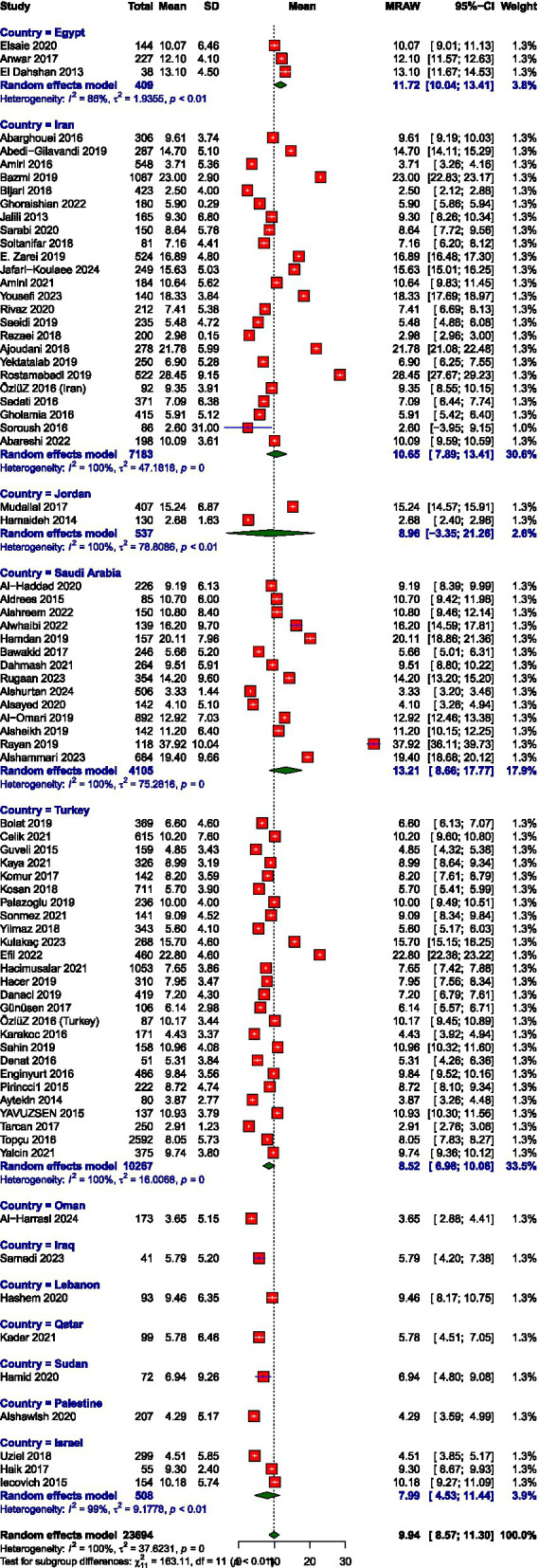
Mean score of depersonalization.

**Figure 6:**
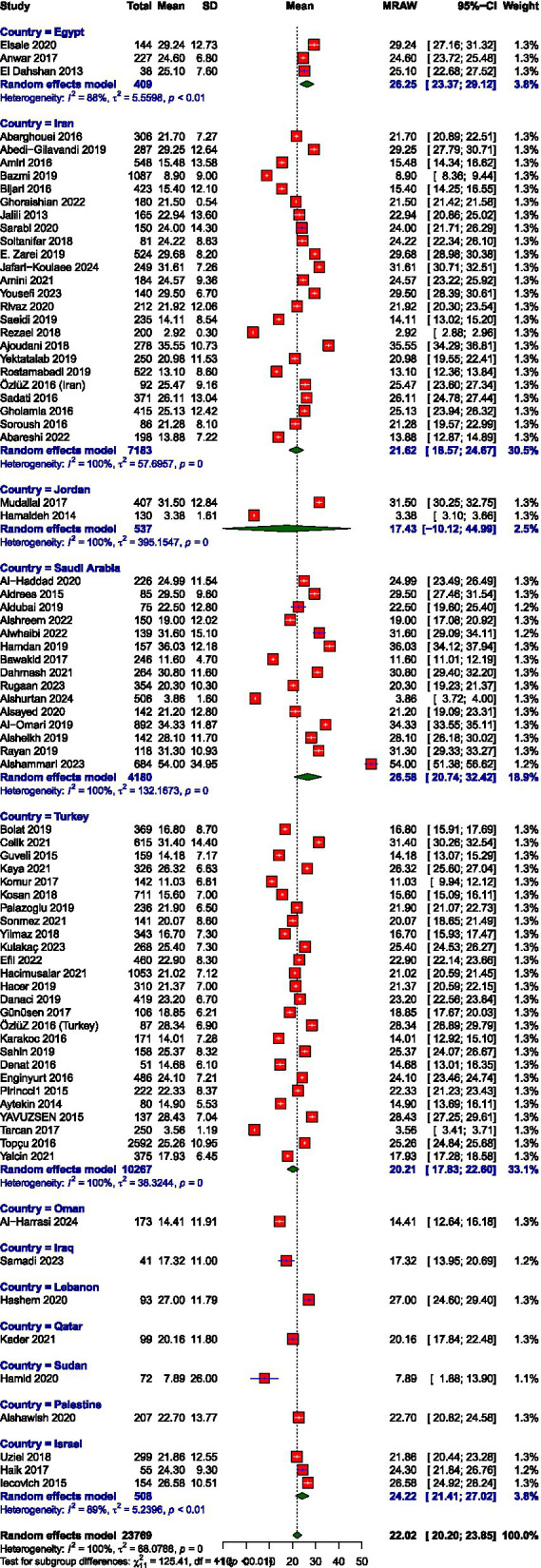
Mean score of emotional exhaustion.

**Figure 7:**
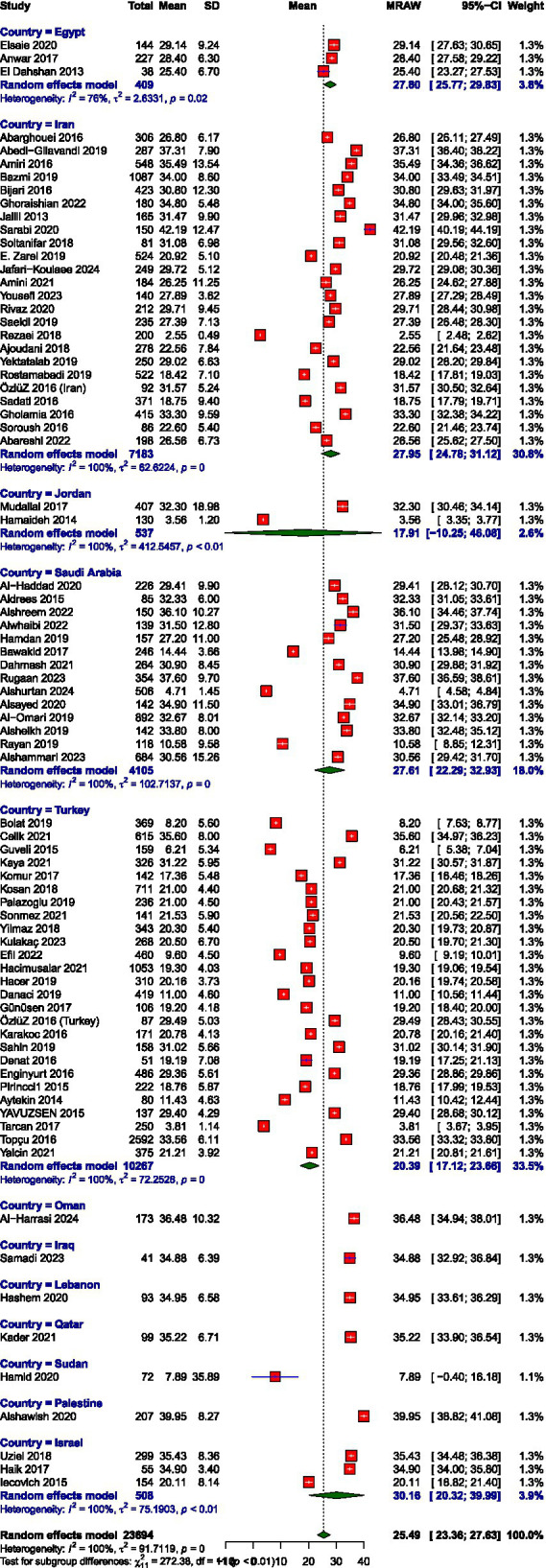
Mean score of personal accomplishment.

The pooled mean scores from the random-effects meta-analysis indicated moderate levels of burnout across the EE and DP and high PA subscales among healthcare workers in the MENAT region. The average score for EE was 22.02 (95% CI: 20.20–23.85), for DP was 9.93 (95% CI: 8.57–11.30), and for PA was 25.49 (95% CI: 23.36–27.63). Meta-analysis forest plots are presented in Figures 2–7. All three subscales, EE, DP, and PA, demonstrated substantial between-study heterogeneity, *I*^2^ = 100, 99.9, and 100, respectively. Our subgroup analysis of MENAT regions indicated a difference in MBI scores. For EE, Sudan (7.89) and Oman (14.41) demonstrated the lowest levels, indicating little burnout, followed by moderate levels in Iraq (17.32), Jordan (17.43), Qatar (20.16), Turkey (20.21), Iran (21.62), Palestine (22.70), and Israel (24.22). Higher EE rates were seen in Egypt (26.25), Saudi Arabia (26.58), and Lebanon (27.00). Regarding depersonalization (DP), the lowest scores were recorded in Oman (3.65), Palestine (4.29), Qatar (5.78), Iraq (5.79), and Sudan (6.94). Israel (7.99), Turkey (8.52), Jordan (8.96), Lebanon (9.46), and Iran (10.65) exhibited moderate DP levels, but elevated levels were observed in Egypt (11.72) and Saudi Arabia (13.21). Finally, the PA subscale, where lower scores represent increased burnout, showed Sudan (7.89), Jordan (17.91), and Turkey (20.39) with the lowest PA, suggesting greater burnout. Moderate PA ratings were reported in Egypt (27.61), Iran (27.95), and Israel (30.16). In contrast, higher PA scores were recorded in Iraq (34.88), Lebanon (34.95), Qatar (35.22), Oman (36.48), and Palestine (39.95), the last showing the highest sense of personal accomplishment among the countries analyzed (Table 2).

**Table 2:** Mean scores on burnout subscales.

Domain of burnout	Country	Number of studies	Mean	95%-CI (%)	I2
EE score	**Overall**	**79**	**22.02**	**[20.19; 23.85]**	**100**
	Egypt	3	26.25	[23.37; 29.12]	87.7
	Iran	24	21.62	[18.57; 24.67]	100
	Jordan	2	17.43	[−10.12; 44.99]	99.9
	Saudi Arabia	15	26.58	[20.74; 32.42]	99.9
	Turkey	26	20.21	[17.83; 22.60]	99.9
	Oman	1	14.41	[12.64; 16.18]	--
	Iraq	1	17.32	[13.95; 20.69]	--
	Lebanon	1	27	[24.60; 29.40]	--
	Qatar	1	20.16	[17.84; 22.48]	--
	Sudan	1	7.89	[1.88; 13.90]	--
	Palestine	1	22.7	[20.82; 24.58]	--
	Israel	3	24.22	[21.41; 27.02]	88.9
DP score	**Overall**	**78**	**9.93**	**[8.57; 11.30]**	**99.9**
	Egypt	3	11.72	[10.04; 13.41]	86.4
	Iran	24	10.65	[7.89; 13.41]	100
	Jordan	2	8.96	[−3.35; 21.26]	99.9
	Saudi Arabia	14	13.21	[8.66; 17.77]	99.8
	Turkey	26	8.52	[6.98; 10.06]	99.8
	Oman	1	3.65	[2.88; 4.41]	--
	Iraq	1	5.79	[4.20; 7.38]	--
	Lebanon	1	9.46	[8.17; 10.75]	--
	Qatar	1	5.78	[4.51; 7.05]	--
	Sudan	1	6.94	[4.80; 9.08]	--
	Palestine	1	4.29	[3.59; 4.99]	--
	Israel	3	7.99	[4.53; 11.44]	98.6
PA score		78	25.49	[23.36; 27.62]	100
	Egypt	3	27.8	[25.77; 29.83]	76
	Iran	24	27.95	[24.78; 31.12]	100
	Jordan	2	17.91	[−10.25; 46.08]	99.9
	Saudi Arabia	14	27.61	[22.29; 32.93]	99.9
	Turkey	26	20.39	[17.12; 23.66]	100
	Oman	1	36.48	[34.94; 38.02]	--
	Iraq	1	34.88	[32.92; 36.84]	--
	Lebanon	1	34.95	[33.61; 36.29]	--
	Qatar	1	35.22	[33.90; 36.54]	--
	Sudan	1	7.89	[−0.40; 16.18]	--
	Palestine	1	39.95	[38.82; 41.08]	--
	Israel	3	30.16	[20.32; 39.99]	99.5

### Prevalence of burnout subscales

Prevalence estimates were high EE, high DP, and low PA. The pooled prevalence values were high EE: 39.74% (95% CI: 34.61 to 45.10%), high DP: 31.31% (95% CI: 26.36 to 36.72%), and low PA: 38.44% (95% CI: 32.15 to 45.14%). The prevalence of burnout across all three subscales, EE, DP, and diminished PA, demonstrated substantial between-study heterogeneity, I^2^ = 98, 98.1, and 97.6, respectively.

The prevalence of high EE varied remarkably across MENAT region countries. The rates vary through Iraq (17.65%), Oman (17.89%), Iran (27.26%), Israel (27.68%), Lebanon (29.83%), Qatar (33.1%), Bahrain (43.28%), Saudi Arabia (44.86%), Turkey (45.15%), Egypt (47.14%), Morocco (51.11%), Jordan (53.5%), Palestine (54.5%), Sudan (58.89%), and Tunisia (65.71%). The prevalence of DP also showed notable variation across MENAT countries. Lebanon reported the lowest prevalence at 11.27%, followed by Israel (17.31%) and Iraq (17.65%), Qatar (20.24%), Iran (23.98%), Bahrain (26.87%), Sudan (31.4%), Palestine (32.68%), Turkey (33.57%), Egypt (34.48%), Jordan (35.13%), Oman (37.89%), Saudi Arabia (39.03%), Morocco (42.88%), and Tunisia (50%), reflecting a considerable burden of detachment and cynicism in these populations. The prevalence of low PA varied widely across MENAT countries: Oman (21.05%), Sudan (21.39%), Morocco (23.54%), Israel (27.97%), Saudi Arabia (29.88%), Lebanon (30.61%), Qatar (31.99%), Egypt (32.14%), Iraq (41.18%), Tunisia (45.71%), Iran (48.15%), Bahrain (51.74%), Turkey (54.06%), Jordan (63.82%), and Palestine (65.63%) (Table 3).

**Table 3:** Prevalence of burnout subscales.

Domains of Burnout	Country	Number of studies	Prevalence (%)	95%-CI (%)	I2 (%)
High EE	**Overall**	**81**	**39.74**	**[34.61; 45.10]**	**98**
	Egypt	5	47.14	[31.26; 63.61]	95.9
	Iran	20	27.26	[19.77; 36.29]	97.4
	Jordan	2	53.5	[41.98; 64.66]	91
	Saudi Arabia	26	44.86	[35.87; 54.21]	97
	Turkey	9	45.15	[25.88; 65.99]	98.8
	Tunisia	1	65.71	[53.92; 75.84]	—
	Morocco	4	51.11	[42.35; 59.80]	73.1
	Iraq	1	17.65	[8.15; 34.10]	—
	Sudan	2	58.89	[39.91; 75.54]	93.5
	Qatar	2	33.1	[18.23; 52.33]	94.8
	Oman	1	17.89	[13.07; 24.00]	—
	Lebanon	3	29.83	[21.63; 39.57]	90.1
	Bahrain	1	43.28	[36.60; 50.22]	—
	Palestine	2	54.5	[30.37; 76.69]	97.9
	Israel	2	27.68	[23.27; 32.58]	34.2
High DP	**Overall**	**81**	**31.31**	**[26.36; 36.72]**	**98.1**
	Egypt	5	34.48	[22.27; 49.15]	96.9
	Iran	20	23.98	[14.53; 36.91]	98.7
	Jordan	2	35.13	[8.64; 75.63]	98.8
	Saudi Arabia	26	39.03	[31.54; 47.08]	97.7
	Turkey	9	33.57	[17.64; 54.37]	98.8
	Tunisia	1	50	[38.50; 61.50]	—
	Morocco	4	42.88	[39.44; 46.38]	67.5
	Iraq	1	17.65	[8.15; 34.10]	—
	Sudan	2	31.4	[16.57; 51.33]	92.4
	Qatar	2	20.24	[12.02; 32.05]	87.6
	Oman	1	37.89	[31.28; 44.99]	—
	Lebanon	3	11.27	[9.58; 13.22]	0
	Bahrain	1	26.87	[21.19; 33.41]	—
	Palestine	2	32.68	[9.61; 68.92]	98.7
	Israel	2	17.31	[9.42; 29.65]	87.9
Low PA	**Overall**	**81**	**38.44**	**[32.15;45.14]**	**97.6**
	Egypt	5	32.14	[5.11; 80.66]	98.7
	Iran	20	48.15	[36.46; 60.05]	96.8
	Jordan	2	63.82	[25.56; 90.06]	98.7
	Saudi Arabia	26	29.88	[21.54; 39.80]	97.7
	Turkey	9	54.06	[37.56; 69.71]	98.9
	Tunisia	1	45.71	[34.48; 57.41]	—
	Morocco	4	23.54	[10.53; 44.62]	95.9
	Iraq	1	41.18	[26.12; 58.09]	—
	Sudan	2	21.39	[5.77; 54.75]	97.4
	Qatar	2	31.99	[20.08; 46.83]	91.9
	Oman	1	21.05	[15.84; 27.43]	—
	Lebanon	3	30.61	[15.35; 51.75]	97.4
	Bahrain	1	51.74	[44.84; 58.57]	—
	Palestine	2	65.63	[60.95; 70.03]	31.1
	Israel	2	27.97	[23.54; 32.87]	0

### Publication bias

Visual inspection of funnel plots and Egger’s tests for EE prevalence and DP prevalence revealed evidence of asymmetry in prevalence values, *p* = 0.034 and 0.002, respectively. But for diminished PA prevalence, we detected no funnel plot asymmetry between *p* = 0.74.

### Determinants of burnout

It was not feasible to synthesize the results using meta-analytic techniques due to substantial heterogeneity in the assessment of determinants and the reporting of outcomes across studies. Notably, several components of the work environment demonstrated statistically significant associations with measures of burnout, including heavy workload, unstructured and demanding working conditions, challenges in maintaining a work-life balance, and income-related stress. For the purpose of this review, these determinants have been systematically categorized into two overarching domains: individual factors and work-related factors.

### Individual factors

A positive relationship between age and burnout was reported by several studies (Bijari and Abassi, 2016; Elsaie et al., 2020; Rugaan et al., 2023; Zarei et al., 2019). A negative correlation between age and emotional exhaustion was reported by three studies (Elsaie et al., 2020; Kosan et al., 2018; Sonmez and Gul, 2021). Multiple studies reported a positive relationship between age and rating higher on the personal accomplishment sub-scale (Al-Haddad et al., 2020; Bijari and Abassi, 2016; Rugaan et al., 2023; Zarei et al., 2019). The findings on the relationship between gender and burnout dimensions were inconsistent.

There was a significant difference between the frequency of nurses’ emotional exhaustion domain in terms of gender, women suffered more from emotional exhaustion (Assadi et al., 2019). Female nurses were significantly more likely to experience burnout (Rugaan et al., 2023). Female family medicine residents were found to have a significantly (*p* = 0.002) higher EE and significantly lower PA than males (AlNahedh et al., 2023).

There was significant relationship between marital status and depersonalization (Abedi-Gilavandi et al., 2019), also emotional exhaustion rates were found to be higher in married individuals (Aldrees et al., 2015). Burnout was found to be significantly higher in individuals having more than three children (Bijari and Abassi, 2016).

### Work-related factors

Increased workload/ high caseloads were found consistently by the studies in this review to be associated with higher rates of burnout (Aldubai et al., 2019; Alshreem et al., 2022; Celik et al., 2021; Elsaie et al., 2020; Ghoraishian et al., 2022; Jalili et al., 2013; Kosan et al., 2018; Rugaan et al., 2023; Shbeer and Ageel, 2022). Increasing workload were associated with higher rates of BO in oncologists (Abusanad et al., 2021).

A sense of autonomy at work and perceived capacity to influence decisions that affect work was reported by one study identified in this review to be associated with lower rates of burnout, particularly increased rates of professional accomplishment and lower rate of emotional exhaustion (Mudallal et al., 2017).

Patient pressure and violence were reported as significant predictors of burnout (Bawakid et al., 2017). Personal accomplishment and depersonalization were found to be higher in individuals working in rural areas as compared to urban areas (Elsaie et al., 2020). Working in public hospitals was associated with increased levels of burnout (Ghoraishian et al., 2022; Kosan et al., 2018).

## Discussion

This review included data on the prevalence and determinants of burnout among healthcare workers (HCWs) from 123 studies across the MENAT region. The updated pooled mean scores for the three MBI-HSS subscales were 22.02 for emotional exhaustion, 10.07 for depersonalization, and 25.49 for personal accomplishment. These means indicate that the average HCW experiences a ‘moderate’ level of emotional exhaustion and depersonalization but maintains a ‘high’ level of personal accomplishment. This finding suggests that HCWs may still feel competent and effective despite experiencing exhaustion, overextension, depletion, and disconnection.

The prevalence estimates for the burnout dimensions were 40% for emotional exhaustion, 31% for depersonalization, and 38% for a low sense of personal accomplishment. Comparatively, studies from Western Europe and North America report emotional exhaustion rates ranging from 19 to 42%, depersonalization from 17 to 34%, and low personal accomplishment from 20 to 44% (Hiver et al., 2022; West et al., 2016). While emotional exhaustion and depersonalization levels in MENAT HCWs are comparable to the upper limits of those observed in Western HCWs, the prevalence of low personal accomplishment is significantly higher. This suggests that despite facing similar levels of exhaustion and depersonalization, HCWs in the MENAT region perceive their professional efficacy to be lower than their Western counterparts which indicates a unique regional concern.

Several factors may contribute to the higher prevalence of low personal accomplishment in MENAT HCWs. The healthcare infrastructure in many MENAT countries is often under-resourced, with HCWs managing high patient loads and administrative burdens while lacking adequate institutional support, limiting their sense of achievement. Moreover, career progression pathways in the region are less structured compared to Western settings, where mentorship programs, skill development opportunities, and performance-based incentives reinforce professional growth. Additionally, hierarchical workplace structures common in MENAT countries may restrict autonomy, with junior staff having limited influence over clinical decisions, which contrasts with the more collaborative environments seen in Western healthcare systems. Sociocultural norms also play a role, as mental health stigma remains prevalent, discouraging HCWs from seeking psychological support, further exacerbating stress and diminishing professional fulfillment.

The relationship between age and burnout in MENAT HCWs varied across studies. Some findings suggest that older HCWs experience lower burnout levels, possibly due to greater experience, coping strategies, and professional stability (Bijari and Abassi, 2016; Elsaie et al., 2020). However, other studies indicate that burnout may increase with age, potentially due to prolonged exposure to occupational stressors (Rugaan et al., 2023; Zarei et al., 2019). Similarly, gender differences in burnout were inconsistent across studies. Some findings indicate that female HCWs, particularly nurses and medical residents, experience higher emotional exhaustion and lower personal accomplishment (Assadi et al., 2019; Rugaan et al., 2023). This could be attributed to increased caregiving responsibilities, fewer leadership opportunities, and gender disparities in workplace autonomy. However, other studies found no significant gender differences in burnout prevalence (Chemali et al., 2019). The inconsistency in findings suggests that gender-related burnout patterns may be influenced by specialty, workplace environment, and cultural expectations.

The findings of this systematic review and meta-analysis align with previous studies in the MENAT region, which have shown that burnout is prevalent among physicians, nurses, and other healthcare professionals (Abusanad et al., 2022; Chemali et al., 2019). Previous research has reported high burnout rates with some studies showing even higher estimates (Dubale et al., 2019; Elbarazi et al., 2017), particularly in conflict-affected regions (Aldabbour et al., 2025). High burnout levels have been associated with workload pressures, limited resources, and exposure to violence in healthcare settings. Compared to Western HCWs (Hiver et al., 2022), MENAT HCWs report higher levels of low personal accomplishment, which may be a reflection of systemic workplace challenges, career stagnation, and insufficient professional recognition. These findings underscore the urgent need for policy reforms, structured professional development, and institutional support systems to reduce burnout and enhance job satisfaction among HCWs in the MENAT region.

This review has several limitations. One key limitation is that all included studies were cross-sectional, which limits the ability to establish causal relationships between risk and protective factors and burnout. While cross-sectional designs are appropriate for estimating burnout prevalence, they only allow for correlational interpretations of factors contributing to burnout. As a result, the associations between workload, autonomy, gender, and burnout should be interpreted with caution, as causal inferences cannot be drawn. Additionally, the included studies demonstrated substantial heterogeneity, a common characteristic of meta-analyses on prevalence data, particularly in healthcare research (Hodkinson et al., 2022; Rotenstein et al., 2018), To mitigate this, we applied strict inclusion criteria, focusing exclusively on studies using the MBI-HSS and reporting burnout by standardized subscales. Despite these efforts, the consistently high *I*^2^ values across all three burnout dimensions highlight significant variability in study populations, settings, and assessment methods. This level of heterogeneity may impact the generalizability and interpretability of the pooled prevalence estimates. However, sensitivity analyses indicated that studies with higher methodological rigor tended to report slightly lower burnout prevalence. This pattern suggests that, despite the presence of heterogeneity, the key findings of this review remain robust and reflective of underlying trends.

Another limitation is that burnout assessments relied primarily on self-reported measures, which are subject to recall bias and social desirability bias. Future research should integrate objective measures, such as stress biomarkers or longitudinal tracking of burnout trends, to complement self-reported data. While this review provides a comprehensive overview of burnout across the MENAT region; however, data were lacking from several countries, including the United Arab Emirates, Kuwait, Libya, Syria, and Yemen. This gap limits the regional representativeness of the findings and underscores the need for future research in these underrepresented and potentially high-burden contexts. While our findings reflect broad regional trends, more country-specific investigations are essential to uncover context-specific risk factors and to inform targeted, locally appropriate intervention strategies.

Future research should explore context-specific burnout mitigation strategies tailored to the MENAT region, as many existing interventions have been tested in Western healthcare settings and may not be directly transferable. Longitudinal studies tracking burnout trends over time and intervention effectiveness would provide stronger evidence for policy recommendations and workplace interventions. Addressing burnout requires a multi-level approach, involving healthcare organizations, policymakers, and individual-level interventions, including workplace wellness programs, improved workload management, and psychological support services. Given the role of mental health stigma in the region, awareness campaigns and institutional policies normalizing psychological support-seeking behaviors could be key to burnout prevention efforts.

## Data Availability

All data underlying the results of this study are available within the article and its supplementary materials. The study used data extracted from previously published articles, which are publicly accessible through their respective journals and databases.
