# Supplementary material for: Prevalence of burnout and its risk and protective factors among healthcare workers in the Middle East, North Africa, and Turkey: a systematic review and meta-analysis
Source: Front Psychol. 2025 Oct 22;16:1539105. doi: 10.3389/fpsyg.2025.1539105 (PMC12586964; doi:10.3389/fpsyg.2025.1539105)
Supplement: Supplementary file 1 [file Table_1.docx]

**Prevalence of Burnout and Its Risk and Protective Factors Among Healthcare Workers in the Middle East, North Africa, and Turkey: A Systematic Review and Meta-Analysis**

Search Strategy:

( "burnout" OR "occupational burnout" OR "occupational stress" OR "work-related stress" OR "emotional exhaustion" OR "job stress" OR "professional burnout" )

AND

( "healthcare workers" OR "healthcare personnel" OR "health professionals" OR "medical staff" OR "health staff" OR "nurse" OR "nurses" OR "doctor" OR "doctors" OR "physician" OR "physicians" OR "clinicians" OR "healthcare providers" )

AND

( "Middle East" OR "North Africa" OR "Turkey" OR "MENA" OR "MENAT" OR "Arab countries" OR "Arab world" OR "Maghreb" OR "Levant" OR "Gulf Cooperation Council" OR "GCC" OR "Arabian Peninsula" OR "Algeria" OR "Bahrain" OR "Egypt" OR "Iran" OR "Iraq" OR "Israel" OR "Jordan" OR "Kuwait" OR "Lebanon" OR "Libya" OR "Morocco" OR "Oman" OR "Palestine" OR "Qatar" OR "Saudi Arabia" OR "Sudan" OR "Syria" OR "Tunisia" OR "United Arab Emirates" OR "UAE" OR "Yemen" )
