# Supplementary material for: Prevalence of burnout and its risk and protective factors among healthcare workers in the Middle East, North Africa, and Turkey: a systematic review and meta-analysis
Source: Front Psychol. 2025 Oct 22;16:1539105. doi: 10.3389/fpsyg.2025.1539105 (PMC12586964; doi:10.3389/fpsyg.2025.1539105)
Supplement: Supplementary file 2 [file Table_2.docx]

| Study ID | Was the sample frame appropriate to address the target population? | Were study participants sampled in an appropriate way? | Was the sample size adequate? | Were the study subjects and the setting described in detail? | Was the data analysis conducted with sufficient coverage of the identified sample? | Were valid methods used for the identification of the condition? | Was the condition measured in a standard, reliable way for all participants? | Was there appropriate statistical analysis? | Was the response rate adequate, and if not, was the low response rate managed appropriately? |
| --- | --- | --- | --- | --- | --- | --- | --- | --- | --- |
| Elsaie 2020 | Yes | Yes | No | No | Yes | Yes | Yes | Yes | Unclear |
| Abarghouei 2016 | Yes | Yes | Yes | Yes | Yes | Yes | Yes | Yes | Yes |
| Abedi-Gilavandi 2019 | Yes | Yes | Yes | No | Yes | Yes | Yes | Yes | Yes |
| Amiri 2016 | Yes | Yes | Yes | Yes | Yes | Yes | Yes | Yes | Yes |
| Bazmi 2019 | Yes | Yes | Yes | Yes | Yes | Yes | Yes | Yes | Yes |
| Bijari 2016 | Yes | Yes | Yes | Yes | Yes | Yes | Yes | Yes | Yes |
| Ghoraishian 2022 | Yes | Yes | Yes | Yes | Yes | Yes | Yes | Yes | Yes |
| Jalili 2013 | Yes | Yes | Yes | Yes | Yes | Yes | Yes | Yes | Yes |
| Sarabi 2020 | Yes | Yes | Yes | No | Yes | Yes | Yes | Yes | Yes |
| Soltanifar 2018 | Yes | Yes | Yes | Yes | Yes | Yes | Yes | Yes | Yes |
| E. Zarei 2019 | Yes | Yes | Yes | Yes | Yes | Yes | Yes | Yes | Yes |
| Mudallal 2017 | Yes | Yes | Yes | Yes | Yes | Yes | Yes | Yes | Yes |
| Al-Haddad 2020 | Yes | Yes | Yes | No | Yes | Yes | Yes | Yes | Yes |
| Aldrees 2015 | Yes | Yes | Yes | Yes | Yes | Yes | Yes | Yes | Yes |
| Aldubai 2019 | Yes | Yes | Yes | No | Yes | Yes | Yes | Yes | Yes |
| Alshreem 2022 | Yes | Yes | Yes | Yes | Yes | Yes | Yes | Yes | Yes |
| Alwhaibi 2022 | Yes | Yes | Yes | No | Yes | Yes | Yes | Yes | Yes |
| Hamdan 2019 | Yes | Yes | Yes | Yes | Yes | Yes | Yes | Yes | Yes |
| Bawakid 2017 | Yes | Yes | Yes | Yes | Yes | Yes | Yes | Yes | Yes |
| Dahmash 2021 | Yes | Yes | Yes | No | Yes | Yes | Yes | Yes | Yes |
| Rugaan 2023 | Yes | Yes | Yes | No | Yes | Yes | Yes | Yes | Yes |
| Bolat 2019 | Yes | Yes | Yes | Yes | Yes | Yes | Yes | Yes | Yes |
| Celik 2021 | Yes | Yes | Yes | Yes | Yes | Yes | Yes | Yes | Yes |
| Guveli 2015 | Yes | Yes | Yes | Yes | Yes | Yes | Yes | Yes | Yes |
| Kaya 2021 | Yes | Yes | Yes | Yes | Yes | Yes | Yes | Yes | Yes |
| Komur 2017 | Yes | Yes | Yes | Yes | Yes | Yes | Yes | Yes | Yes |
| Kosan 2018 | Yes | Yes | Yes | Yes | Yes | Yes | Yes | Yes | Yes |
| Palazoglu 2019 | Yes | Yes | Yes | Yes | Yes | Yes | Yes | Yes | Yes |
| Sonmez 2021 | Yes | Yes | Yes | Yes | Yes | Yes | Yes | Yes | Yes |
| Yilmaz 2018 | Yes | Yes | Yes | No | Yes | Yes | Yes | Yes | Yes |
| [Azeez 2024](https://oss.signavitae.com/mre-signavitae/article/20241108-465/pdf/SV2024032001.pdf) | Yes | Yes | Yes | Yes | Yes | Yes | Yes | Yes | Yes |
| [Hassan 2024](https://bmcpsychiatry.biomedcentral.com/counter/pdf/10.1186/s12888-024-06196-y.pdf) | Yes | Yes | Yes | Yes | Yes | Yes | Yes | Yes | Unclear |
| [Zemni 2024](https://onlinelibrary.wiley.com/doi/pdfdirect/10.1111/jep.14021) | Yes | Yes | Yes | Yes | Yes | Yes | Yes | Yes | Unclear |
| [Jafari-Koulaee 2024](https://pubmed.ncbi.nlm.nih.gov/38860949/) | Yes | Yes | Unclear | Yes | Yes | Yes | Yes | Yes | Unclear |
| [Hamdan 2023](https://bmcmededuc.biomedcentral.com/counter/pdf/10.1186/s12909-023-04572-y.pdf) | Yes | No | Yes | Yes | Yes | Yes | Yes | Yes | Unclear |
| [Al-Harrasi 2024](https://pubmed.ncbi.nlm.nih.gov/38758275/#full-view-affiliation-1) | Yes | No | Unclear | Yes | No | Yes | Yes | Yes | Yes |
| [Alshurtan 2024](https://pmc.ncbi.nlm.nih.gov/articles/PMC10908335/pdf/jmdh-17-843.pdf) | Yes | Yes | Yes | Yes | Yes | Yes | Yes | Yes | Unclear |
| [Alenezi 2022](https://pmc.ncbi.nlm.nih.gov/articles/PMC9590646/pdf/ap-23-4-173.pdf) | Yes | Yes | Yes | Yes | Yes | Yes | Yes | Yes | Yes |
| [Kulakaç 2023](https://pubmed.ncbi.nlm.nih.gov/37269273/) | Yes | Yes | Yes | Yes | Yes | Yes | Yes | Yes | Yes |
| [Kasemy 2023](https://bmjopen.bmj.com/content/bmjopen/13/10/e074645.full.pdf) | Yes | Unclear | Yes | Yes | Yes | Yes | Yes | Yes | Yes |
| [Amini 202](https://pubmed.ncbi.nlm.nih.gov/35146848/)1 | Yes | Yes | Yes | Yes | Yes | Yes | Yes | Yes | Yes |
| [Chahbounia 2023](https://pmc.ncbi.nlm.nih.gov/articles/PMC10443326/pdf/nursrep-13-00094.pdf) | Yes | Yes | Yes | Yes | Yes | Yes | Yes | Yes | Yes |
| [Al Sabei 2023](https://pubmed.ncbi.nlm.nih.gov/37515995/#full-view-affiliation-1) | Yes | No | Yes | Yes | Unclear | Yes | Yes | Yes | Yes |
| [Chtibi 2023](https://rediviva.sav.sk/65i4/139.pdf) | Yes | No | Unclear | Yes | Yes | Yes | Yes | Yes | Unclear |
| [Ebrahimpour 2023](https://pmc.ncbi.nlm.nih.gov/articles/PMC10167527/pdf/ABJS-11-293.pdf) | Yes | No | Yes | Yes | Yes | Yes | Yes | Yes | No |
| [Samadi 2023](https://pmc.ncbi.nlm.nih.gov/articles/PMC9954617/pdf/brainsci-13-00251.pdf) | Yes | Yes | Yes | Yes | Yes | Yes | Yes | Yes | Yes |
| [Yousefi 2023](https://hdq.uswr.ac.ir/article-1-436-en.pdf) | Yes | Yes | Yes | Yes | Yes | Yes | Yes | Yes | Yes |
| [Al‑Ghamdi 2021](https://pmc.ncbi.nlm.nih.gov/articles/PMC8208182/pdf/JFMPC-10-1904.pdf) | Yes | Yes | Yes | Yes | Yes | Yes | Yes | Yes | Yes |
| [Benhamza 2023](https://pmc.ncbi.nlm.nih.gov/articles/PMC10561900/pdf/cureus-0015-00000044956.pdf) | Yes | No | Yes | Yes | Yes | Yes | Yes | Yes | Yes |
| [Alzahrani 2023](https://pmc.ncbi.nlm.nih.gov/articles/PMC10015567/pdf/JMedLife-16-277.pdf) | NO | NO | Unclear | Yes | Yes | Yes | Yes | Yes | Unclear |
| [Qedair 2022](https://pmc.ncbi.nlm.nih.gov/articles/PMC9607756/pdf/12912_2022_Article_1070.pdf) | Yes | NO | Yes | Yes | Yes | Yes | Yes | Yes | NO |
| [Ghazwani 2022](https://pmc.ncbi.nlm.nih.gov/articles/PMC8810484/pdf/fpubh-09-834407.pdf) | Yes | Yes | Yes | Yes | Yes | Yes | Yes | Yes | Yes |
| [Elhadi 2022](https://pmc.ncbi.nlm.nih.gov/articles/PMC8896711/pdf/pone.0265098.pdf) | Yes | Yes | Unclear | Yes | Yes | Yes | Yes | Yes | Yes |
| [Alqarni1 2022](https://pmc.ncbi.nlm.nih.gov/articles/PMC9045659/pdf/pone.0267578.pdf) | Yes | Yes | Unclear | Yes | Yes | Yes | Yes | Yes | Yes |
| [Efıl 2022](https://pubmed.ncbi.nlm.nih.gov/35397980/#full-view-affiliation-1) | Unclear | NO | Yes | Yes | Yes | Yes | Unclear |  |  |
| [Ibrahim 2022](https://pmc.ncbi.nlm.nih.gov/articles/PMC8941313/pdf/jphr-11-2-2436.pdf) | Yes | Yes | Yes | Yes | Yes | Yes | Yes | Yes | Yes |
| [AlHadi 2022](https://journals.lww.com/jnsm/fulltext/2022/05010/prevalence_and_treatment_preference_of_burnout,.12.aspx) | Yes | No | Yes | Yes | Yes | Yes | Yes | Yes | No |
| [El-Menyar 2020](https://www.sci-hub.st/downloads/2020-10-27/19/el-menyar2020.pdf) | Yes | Yes | Yes | Yes | Yes | Yes | Yes | Yes | Yes |
| [Hacimusalar 2021](https://pmc.ncbi.nlm.nih.gov/articles/PMC8600578/pdf/MDL-112-346.pdf) | No | No | Unclear | Yes | Yes | Yes | Yes | Yes | No |
| [Yazıcı 2019](https://www.sci-hub.st/10.1097/PEC.0000000000001839) | Yes | Yes | Unclear | Yes | Yes | Yes | Yes | Yes | Yes |
| [Alsayed 2020](https://ejcm.journals.ekb.eg/article_187673_dcf1a3670cc1b7d4dcf137ebfcde685c.pdf) | Yes | No | No | Yes | Yes | Yes | Yes | Yes | Yes |
| [Almodibeg 2020](https://pmc.ncbi.nlm.nih.gov/articles/PMC7729769/pdf/NOP2-8-364.pdf) | Yes | Yes | Yes | Yes | Yes | Yes | Yes | Yes | Yes |
| [Biganeh 2021](https://www.jstage.jst.go.jp/article/juoeh/43/4/43_397/_pdf) | Yes | Yes | Yes | Yes | Yes | Yes | Yes | Yes | Unclear |
| [Hashem 2020](https://www.sci-hub.st/10.1007/s12671-020-01469-5) | No | No | No | Yes | Yes | Yes | Yes | Yes | No |
| [Soltan 2020](https://mecp.springeropen.com/counter/pdf/10.1186/s43045-020-00046-9.pdf) | Yes | No | Unclear | Yes | Yes | Yes | Yes | Yes | Unclear |
| [Kader 2021](https://pdf.sciencedirectassets.com/277359/1-s2.0-S1876201821X00032/1-s2.0-S1876201821000757/main.pdf?X-Amz-Security-Token=IQoJb3JpZ2luX2VjEJr%2F%2F%2F%2F%2F%2F%2F%2F%2F%2FwEaCXVzLWVhc3QtMSJHMEUCIERU0XmQbnocCj7uBZnvHnhTIeAuVx67%2Fls%2FcJUKcQR5AiEAuaDNAi9lC6qfDwRhYbnfynXjO0ZeIWdwHQzJsfbkaOkqsgUIQxAFGgwwNTkwMDM1NDY4NjUiDGFH1nMde%2FPkDxugKyqPBfFY91bmEt287sIdbc2%2BtBcUR%2BdoBRREa2vcErKC0taKMKi%2FIVFcTUE8sExXoTtNb34b%2BCQhhz2r21BhOxPYBe1sSPnk48QOt2ox%2BHfAkea%2Bh%2Fswrj0%2Fh392L6sS%2Bq5McYv%2FwPvV7LMLVYhMaZpBSi1T3W9OGGnkxJwQiXRIYTHcUQQ9phuz%2BnzCw8TJKjcalMB3lkRAdlWTq4YSsaw7AhwxwedKV2m%2FkCFAz041CVRAEbwApdpbzY3Fxd0QWwTbNUNzFqJn0TrRuEFVxsxkJhyPtNMtz0M5sizQuGbU6rfv%2FEk%2BG2j0mx24mUDkwDd0tUE0vUet5%2FOwrUnLy1sy0hd6uynzEMk9zwGtiUN%2BqNqkQp8qO8uB41lwFq2ElpCggq%2BAwuz0vmDyxWQYyvqM6xyQbW3aysT3dWDjIpg%2BZ8TOhBCOx2yCxkfaiC8zRzytzhemcbvJp%2BbpsrTULGJdtH%2Baf5EkNxU6UlPc0hM9Uth%2B3HDlq9qsPmhZmnBSsyBemfpUvvDclVOl4zNv1QRqXFqyp2UIrhMimPVOa7UkOA0RczAsltaMThxlOQCDEM%2B1G6E%2BN2bXGYCtkjGLad5EeQQTS0%2Bn9mU9aWqXt5SYRVItqPZgrUnNYT6NjizroNxrrpCMk%2FVUTx3eNXMYC38BWGCtQD5xXlUnaCn%2BISNGha3Iz5Ix4keCwi83z0h6FIotPUaI%2Ffxg6XFvUQgkzr22AjRuAnqi1Vf%2BIcqTv8wU%2FgVetcXopDeLSZFk5%2FQShj9GAsKK1vKnKbEem9lJ%2FIaAyvRyY3LP7bVeyEEc%2Ff3WGB738NrJ2KcVH0pwr66cNZLT0dkf%2F0RoBmh%2Fdt5hTQWSekCSbUxUCLKCszNokwi2qHgw0rznwAY6sQFygeQtwReYkU3EwGDE9REHxTTXJYN7MXSVQFRJqVthczBE5PdJP6SQki14kmD7UM92tg8LL25LBT9j%2BqDatBnec%2FNjLkQGL8vC0cCByQoSjtBLxWQXXhwmuuorelyKmP6z05XSNiY%2FlZze26jR6F1r%2B0tzuS76mX%2FH%2BYvyaJLNVWHBQb%2B%2BeTFZPtWQamhz7l3erHPnmCyRpyacEcv%2BqAl1tys3Y31vjRdTjzRAhZ82SOA%3D&X-Amz-Algorithm=AWS4-HMAC-SHA256&X-Amz-Date=20250506T111025Z&X-Amz-SignedHeaders=host&X-Amz-Expires=300&X-Amz-Credential=ASIAQ3PHCVTYX4EUHUFC%2F20250506%2Fus-east-1%2Fs3%2Faws4_request&X-Amz-Signature=e5151dc80e760d5504dd22d415154685772549fb0b459a3c8daae33aeb108b04&hash=15a185b46ec9f01ac681aa4ea9a249cde217410a547928bc9844629fe1b0bc66&host=68042c943591013ac2b2430a89b270f6af2c76d8dfd086a07176afe7c76c2c61&pii=S1876201821000757&tid=spdf-f5a46d87-9ab5-4513-988d-e2d503b0cfd4&sid=bb09ec898a409047ae5a9e13a39cfaef9ae3gxrqb&type=client&tsoh=d3d3LnNjaWVuY2VkaXJlY3QuY29t&rh=d3d3LnNjaWVuY2VkaXJlY3QuY29t&ua=1f015957035b5755060250&rr=93b807ee7af8bac4&cc=eg) | Yes | Yes | Yes | Yes | Yes | Yes | Yes | Yes | Yes |
| [Shahin 2020](https://pmc.ncbi.nlm.nih.gov/articles/PMC7719557/pdf/publichealth-07-04-065.pdf) | Yes | Yes | Unclear | Yes | Yes | Yes | Yes | Yes | Unclear |
| [Al-Omari 2019](https://www.mdpi.com/2076-3417/10/1/157/pdf?version=1578032032) | No | No | Yes | Yes | Yes | Yes | Yes | Yes | Yes |
| [Alshawish 2020](https://sci-hub.se/10.1080/00207411.2020.1752064) | Yes | No | Unclear | Yes | Yes | Yes | Yes | Yes | Yes |
| [Rivaz 2020](https://pmc.ncbi.nlm.nih.gov/articles/PMC7885543/pdf/2216-0280-iee-38-03-e12.pdf) | Yes | Yes | Yes | Yes | Yes | Yes | Yes | Yes | Unclear |
| [Hacer 2019](https://sci-hub.se/10.1016/j.jflm.2019.101874) | No | No | Unclear | Yes | Yes | Yes | Yes | Yes | Unclear |
| [Alotni 2020](https://opennursingjournal.com/VOLUME/14/PAGE/190/FULLTEXT/) | Yes | No | Unclear | Yes | Yes | Yes | Yes | Yes | Yes |
| [Hamid 2020](https://sci-hub.se/10.1177/0081246319898054) | Unclear | Yes | Unclear | Yes | Yes | Yes | Yes | Yes | Unclear |
| [Danaci 2019](https://www.sci-hub.st/downloads/2019-04-11/f1/10.1177@0969733019836151.pdf) | Yes | Yes | Yes | Yes | Yes | Yes | Yes | Yes | Yes |
| [Saeidi 2019](https://journals.lww.com/nams/_layouts/15/oaks.journals/downloadpdf.aspx?trckng_src_pg=ArticleViewer&an=01709572-202009020-00004) | Yes | Yes | Yes | Yes | Yes | Yes | Yes | Yes | Unclear |
| [Abidli 2019](https://rjptonline.org/HTMLPaper.aspx?Journal=Research+Journal+of+Pharmacy+and+Technology%3bPID%3d2019-12-11-15) | No | No | Unclear | Yes | Yes | Yes | Yes | Yes | Yes |
| [Rezaei 2018](https://www.sci-hub.st/10.1515/ijamh-2017-0146) | Yes | Yes | Yes | Yes | Yes | Yes | Yes | Yes | Unclear |
| [Ajoudani 2018](https://dacemirror.sci-hub.st/journal-article/9aa812282f1230b60a6410b61a0df675/ajoudani2018.pdf) | Yes | Yes | Yes | Yes | Yes | Yes | Yes | Yes | Yes |
| [Alqahtani 2019](https://pmc.ncbi.nlm.nih.gov/articles/PMC6398028/pdf/TSWJ2019-4515972.pdf) | Yes | Yes | Unclear | Yes | Yes | Yes | Yes | Yes | Yes |
| [Alsheikh 2019](https://journalmsr.com/content/136/2019/3/2/Images/SaudiOrthopJ_2019_3_2_184_255334.pdf) | Yes | Yes | Unclear | Yes | Yes | Yes | Yes | Yes | No |
| [Yektatalab 2019](https://pmc.ncbi.nlm.nih.gov/articles/PMC6859009/pdf/PAMJ-34-22.pdf) | Yes | Yes | Unclear | Yes | Yes | Yes | Yes | Yes | Yes |
| [Elay 2019](https://www.sci-hub.st/10.15537/smj.2019.9.24520) | Yes | No | Unclear | Yes | Yes | Yes | Yes | Yes | Yes |
| [Al-Hashemi 2019](https://pmc.ncbi.nlm.nih.gov/articles/PMC6505344/pdf/OMJ-D-18-00150.pdf) | Yes | Yes | Yes | Yes | Yes | Yes | Yes | Yes | Yes |
| [Salem 2018](https://pmc.ncbi.nlm.nih.gov/articles/PMC6171388/pdf/APJCP-19-2135.pdf) | No | No | Unclear | Yes | Yes | Yes | Yes | Yes | Yes |
| [Rostamabadi 2019](https://pmc.ncbi.nlm.nih.gov/articles/PMC7809992/pdf/MDL-110-312.pdf) | Yes | Yes | Unclear | Yes | Yes | Yes | Yes | Yes | Yes |
| [Rayan 2019](https://www.sci-hub.st/10.1097/jnr.0000000000000291) | Yes | No | Yes | Yes | unclear | Yes | Yes | Yes | Yes |
| [Günüşen 2017](https://2024.sci-hub.st/6342/950199dd227f2b219bfa1a85291ed92a/10.1177@1043659616689290.pdf) | Yes | No | Unclear | Yes | unclear | Yes | Yes | Yes | No |
| [Alameddine 2017](https://dacemirror.sci-hub.st/journal-article/da44021293c01d816c8fb2a27319856d/alameddine2017.pdf) | Yes | Yes | Yes | Yes | No | Yes | Yes | Yes | Yes |
| [Ashkar 2018](https://scholarworks.aub.edu.lb/bitstream/handle/10938/31024/2018-4340.pdf?sequence=1&isAllowed=y) | Yes | No | Unclear | Yes | No | yes | yes | yes | No |
| [Aldrees 2017](https://pmc.ncbi.nlm.nih.gov/articles/PMC5556300/pdf/SaudiMedJ-38-832.pdf) | Yes | Yes | Yes | Yes | No | Yes | Yes | Yes | No |
| [Schooley 2016](https://pmc.ncbi.nlm.nih.gov/articles/PMC4998861/pdf/medi-95-e02856.pdf) | Yes | Yes | Yes | Yes | Yes | Yes | Yes | Yes | Yes |
| [ÖzlüZ 2016](https://dacemirror.sci-hub.st/journal-article/7895750830024957e238c1b3bdf71420/10.1016@j.jopan.2015.09.012.pdf) (Turkey) | Yes | Yes | Yes | Yes | Yes | Yes | Yes | Yes | Yes |
| [ÖzlüZ 2016](https://dacemirror.sci-hub.st/journal-article/7895750830024957e238c1b3bdf71420/10.1016@j.jopan.2015.09.012.pdf) (Iran) | Yes | Yes | Yes | Yes | Yes | Yes | Yes | Yes | Yes |
| [Karakoc 2016](https://ijkd.org/index.php/ijkd/article/view/2650/881) | Yes | No | Unclear | Yes | Unclear | Yes | Yes | Yes | Yes |
| [Anwar 2017](https://link.springer.com/article/10.1007/s10389-017-0831-2) | Yes | Yes | Yes | Yes | Yes | Yes | Unclear | Yes | Yes |
| [Jafari 2023](https://pmc.ncbi.nlm.nih.gov/articles/PMC10743321/pdf/bet-11-184.pdf) | Yes | No | Yes | Yes | No | Yes | Yes | Yes | Yes |
| [Şahin 2019](https://jag.journalagent.com/kpd/pdfs/KPD_22_2_148_156.pdf) | Yes | Yes | Yes | Yes | Yes | Yes | Yes | Yes | Yes |
| [Alenezi 2022](https://pmc.ncbi.nlm.nih.gov/articles/PMC9590646/pdf/ap-23-4-173.pdf) | Yes | Yes | Yes | Yes | Yes | Yes | Yes | Yes | Yes |
| [Denat 2016](https://www.sci-hub.st/10.12669/pjms.321.8407) | Yes | No | No | Yes | No | Yes | Yes | Yes | Unclear |
| [El Dahshan 2013](https://www.researchgate.net/publication/277871111_The_Effect_of_Structural_and_Psychological_Empowerment_on_Occupational_Burnout_in_Staff_Nurses_Working_in_Shebin_El-Kom_Hospitals_Menoufiya_Governernate_Egypt) | Yes | No | Unclear | Yes | No | Yes | Yes | Yes | No |
| [Hamaideh 2014](https://www.sci-hub.st/10.1111/inm.12000) | Yes | No | Unclear | Yes | No | Yes | Yes | Yes | No |
| [Enginyurt 2016](https://www.sci-hub.st/10.1071/AH14177) | Yes | Yes | Yes | Yes | Yes | Yes | Yes | Yes | Yes |
| [Pirincci1 2015](https://www.tm.mahidol.ac.th/seameo/2015-46-2/18-640918.pdf) | Yes | Yes | Yes | Yes | Yes | Yes | Yes | Yes | Yes |
| [Al-Sareai 2013](https://pubmed.ncbi.nlm.nih.gov/24617120/) | Yes | Yes | Yes | Yes | Yes | Yes | Yes | Yes | Yes |
| [Aytekin 2014](https://www.ajan.com.au/index.php/AJAN/article/view/1601/333) | Yes | Yes | Yes | Yes | Yes | Yes | Yes | Yes | Yes |
| [Sadati 2016](https://brieflands.com/articles/semj-55895) | Yes | Unclear | Yes | Yes | No | Yes | Yes | Yes | Yes |
| [YAVUZŞEN 2015](https://www.jcog.com.tr/pdf/?pdf=9bc44aef3a0154efef65471c7512c3c3) | Yes | No | Unclear | Yes | No | Yes | Yes | Yes | Unclear |
| [Aldrees 2013](https://pmc.ncbi.nlm.nih.gov/articles/PMC6074879/pdf/asm-5-451.pdf) | Yes | Yes | Yes | Yes | Yes | Yes | Yes | Yes | Yes |
| [Agha 2014](https://www.sci-hub.st/10.3233/WOR-141898) | Yes | No | No | Yes | No | Yes | Unclear | Yes | Yes |
| [Gholamia 2016](https://www.sci-hub.st/10.1080/10803548.2016.1147876) | Yes | Yes | Unclear | Yes | Yes | Yes | Yes | Yes | Yes |
| [Tarcan 2017](https://www.sci-hub.st/10.1016/j.apnr.2017.02.011) | Yes | Yes | Yes | Yes | Yes | Yes | Yes | Yes | Yes |
| [Topçu 2016](https://www.sci-hub.st/10.1111/inr.12247) | Yes | Yes | Yes | Yes | Yes | Yes | Yes | Yes | Yes |
| [Soroush 2016](https://pmc.ncbi.nlm.nih.gov/articles/PMC4979268/) | Yes | Yes | Unclear | Yes | Yes | Yes | Yes | Yes | Unclear |
| Abdo 2015 | Yes | Yes | Yes | Yes | Yes | Yes | Yes | Yes | Yes |
| Gashmard 2015 | Yes | Yes | Unclear | Yes | Yes | Yes | Yes | Yes | Yes |
| Sajedian 2023 | Yes | Yes | Unclear | Yes | Yes | Yes | Yes | Yes | Yes |
| Alshammari 2023 | Yes | No | Unclear | Yes | No | Yes | Yes | Yes | Yes |
| Hasan 2015 | Yes | Yes | Yes | Yes | Yes | Yes | Yes | Yes | Yes |
| Yalcin 2021 | Yes | Yes | Yes | Yes | Yes | Yes | Yes | Yes | Yes |
| Abareshi 2022 | Yes | Yes | Yes | Yes | Yes | Yes | Yes | Yes | Yes |
| [Hassan 2024](https://bmcpsychiatry.biomedcentral.com/counter/pdf/10.1186/s12888-024-06196-y.pdf) | Yes | Unclear | Yes | Yes | Yes | Yes | Yes | Yes | Yes |
| Haik 2017 | Yes | Yes | Yes | Yes | Yes | Yes | Yes | Yes | Yes |
| Iecovich 2015 | Yes | Yes | Yes | Yes | Yes | Yes | Yes | Yes | Yes |
